# Prospective Observational Study of Weight-based Assessment of Sodium Supplements on Ultramarathon Performance (WASSUP)

**DOI:** 10.1186/s40798-021-00302-0

**Published:** 2021-02-17

**Authors:** Grant S. Lipman, Tamara Hew-Butler, Caleb Phillips, Brian Krabak, Patrick Burns

**Affiliations:** 1 Department of Emergency Medicine, Stanford University School of Medicine, 900 Welch Rd, Suite #350, Palo Alto, CA 94304 USA; 2 Exercise and Sport Science, College of Education, Wayne State University, Detroit, MI USA; 3 Computational Science, University of Colorado, Boulder, CO USA; 4 Department of Orthopedics and Sports Medicine, University of Washington, Seattle, WA USA

**Keywords:** Electrolytes, Supplements, Performance, Ultramarathon, Running, Endurance, Dehydration, Overhydration, Exercise-associated hyponatremia

## Abstract

**Background:**

Sodium supplements are ubiquitous in endurance running, but their impact on performance has been subjected to much debate. The objective of the study was to assess the effect of sodium supplementation as a weight-based predictor of race performance in ultramarathon runners.

**Methods:**

Prospective observational study during an 80 km (50 mi) stage of a 6-stage 250 km (155 mi) ultramarathon in Chile, Patagonia, Namibia, and Mongolia. Finish line hydration status as measured by weight change, point-of-care serum sodium, and questionnaire provided sodium ingestion categories at 33rd percentile and 66th percentile both for weight-adjusted rate and total sodium consumption, then analyzed for significant relationships to race performance, dysnatremia, and hydration.

**Results:**

Two hundred sixty-six participants were enrolled, with 217 (82%) with complete sodium supplement rate data, 174 (80%) with finish line sodium, and 161 (74%) with both pre-race weights and total sodium ingestion allowing weight-based analysis. Sodium intake ranged from 131–533 mg/h/kg (2–7.2 gm), with no statistically significant impact on pace, race time, or quintile rank. These outcomes did not change when sodium intake was analyzed as a continuous variable or by sub-group analysis of the 109 (68%) normonatremic runners. When controlled for weight-adjusted sodium intake, performance was poorly correlated with hydration (*r* = − 0.152, 95% CI − 0.348–0.057). Dehydrated runners outperformed those overhydrated, with 11% of top 25th percentile finishers dehydrated (versus 2.8% overhydrated), with 3.6 min/km faster pace and time 4.6 h faster finishing time.

**Conclusions:**

No association was found between sodium supplement intake and ultramarathon performance. Dehydrated runners were found to have the best performance. This reinforces the message to avoid overhydration.

**Supplementary Information:**

The online version contains supplementary material available at 10.1186/s40798-021-00302-0.

## Key Points

- Sodium supplement intake was not significantly associated with ultramarathon race pace, time to completion, or finishing rank.
- While performance was poorly correlated with hydration, dehydrated runners significantly outperformed those overhydrated, with a 3.6 min/km faster pace and 4.6 h faster finishing time.
- Sodium supplementation in ultramarathon runners was not associated with ultramarathon performance. Avoidance of overhydration may improve performance.

## Background

The last decade has witnessed a worldwide 345% participation increase in ultramarathons, greater than the 49% increase in marathons [1], with 83–96% of long distance runners regularly ingesting sodium supplements during races [2]. The majority of endurance athletes believe that electrolyte supplementation is necessary to prevent muscle-cramping, nausea, and hyponatremia [3]. However, most studies fail to demonstrate associations between sodium intake and muscle cramps [4], or that supplements are protective against hyponatremia [2, 3, 5–7]. Since fluid consumption during exercise contributes more to hyponatremia than lack of sodium supplementation [8], the potential ergogenic benefits may be eclipsed by the risks of overhydration with sodium solution during endurance events [6, 9].

Serum sodium is a physiologically regulated variable, and sweat production during endurance exercise results in loss of this electrolyte in addition to total body water [10]. Inter-individual sweat sodium rate and composition varies widely, between 10 and 90 mmol/L [11]. The response to sodium over- and under-consumption are largely corrected by adaptive renal mechanisms [12], even in response to very low dietary sodium intakes [12, 13]. Additionally, there is evidence that extra-renal sodium storage and liberation occurs in both soft tissue and bone [14]. During periods of heat stress, it has been suggested that sweat sodium output changes in response to dietary intake [3] and sodium palatability increases [15]. These physiological mechanisms guard sodium homeostasis, which question the presumptive necessity of sodium supplementation during endurance exercise.

Sodium supplementation is targeted in many commercial products such as sports drinks, and included in nutritional sports guidelines [16, 17]. It is commonly believed that electrolyte supplementation is necessary during endurance exercise to maintain optimal fluid homeostasis and prevent performance decline [18, 19]. While total body water losses may impair performance in high intensity exercise [20], this has not been reflected in endurance exercise with larger fluid deficits [21]. There has been scant evidence supporting the performance benefit of sodium supplementation in endurance exercise or finishing status [22, 23]. Studies found no differences in triathlon race time between those taking salt tablets versus no electrolytes [5, 6, 24], no benefit of sodium concentrations on distances run in 4 h [25], or differences in race time in ultramarathon finishers versus small groups of runners with no electrolytes [26]. As only relatively small endurance running studies have investigated the impact of sodium supplementation on performance, an analysis of weight-based sodium ingestion rates in a larger cohort could provide further insight into this debate. The objective of the study was to assess the association of sodium supplementation as a weight-based predictor of race performance in ultramarathon runners. Our hypothesis was that there would be no significant associations between these variables.

## Methods

### Setting

This study was an analyses of data used to study the effect climate on dysnatremia during a prospective observational study during an 80 km (50 mi) 5th stage of a 6-stage 250 km (155 mi) ultramarathon in Chile and Patagonia (2017), and Namibia, Mongolia, and Chile (2018) [7]. All participants were offered the same amount of water for any given day [approximately 1.5 L per 10–12 km (6–7.5 mi)], had to carry all their own gear and at least 2000 kcal/day (verified during registration), and did not receive any food beyond what they carried. As the races were of similar distances and required similar logistical and physical demands, they were combined for analyses [27–29].

### Research Design

All entrants competing in a RacingThePlanet© ultramarathon who could understand English were invited to participate in the study. During pre-race registration, informed consent was obtained and baseline demographics were recorded along with the runner’s sodium supplementation type, rate, and ingestion strategy. The brand of sodium supplement was reviewed by the researchers to confirm the sodium concentration, and the amount of sodium was then calculated by the runner’s supplementation intake rate and official finishing time (Supplementary datasheet). Stanford University School of Medicine institutional review board approved this study and it is in accordance with the ethical standards outlined in the Declaration of Helsinki.

Prior to the start of the 80 km (50 mi) stage of the race, body weight measurements (with shoes and running gear excluding backpack) were obtained with a battery-powered digital scale (SC-505 HoMedics; Commerce Tsp, MI) placed on a solid level surface. Immediately upon completion of the race at the finish line, study participants were re-weighed and an on-site analysis of serum sodium, blood urea nitrogen, and creatinine were obtained by point-of-care i-STAT (Abbott Point of Care, NJ) from finger-tip blood samples before post-race hydration could occur. Sodium intake rates were calculated by dividing the total sodium consumption by individual finishing time. Both the point-of-care device and the digital scale were calibrated prior to taking measurements.

### Participant Characteristics

Participants were stratified into 33rd and 66th percentile based on the median data distribution, with categories of low sodium consumption (< 200 mg/h; < 2.79 mg/h/kg), moderate ( 200–360 mg/h; 2.79 mg/h/kg–4.78 mg/h/kg), or high (> 360 mg/h; > 4.78 mg/h/kg). Sodium consumption was also analyzed as a continuous variable. The following definitions were used: hypernatremia > 145 mmol L−^1^, normonatremia 135 to < 145 mmol L^−1^, and hyponatremia < 135 mmol L^−1^. Hydration status was based upon body weight changes with > 0% body weight change as overhydration, < 0 to − 3% body weight change as euhydration, and < − 3% body weight change as dehydration [30]. The hot or cold climate category for each race was derived using methods fully described previously [7].

### Statistical Analysis

Demographic and performance variables were analyzed for significant relationship to sodium consumption using one-way analysis of variance (ANOVA) for numeric variables and chi-squared for categorical variables. In the case of one-way ANOVA, the null hypothesis was that there was no significant difference between the mean values for performance and demographic variables between the different sodium consumption groups. Similarly, a chi-squared test was used to assess independence of the distribution of categorical variables from the sodium-based grouping. Both the characteristics of athletes and their performance were considered as main effects from the standpoint of sodium consumption. Lastly, Pearson’s product-moment correlation was used to test for linear relationship between average pace and sodium consumption rate. To exclude the possibility that sodium may have imparted a different effect on higher or lower performing athletes, similarly paced runners performance were compared to their sodium consumption using Pearson’s product-moment correlation. This analysis was performed with percentile-based performance groups (top 10%, 11–25%, 26–50%, 51–75%, and below 75%) and using a sliding window method where sodium consumption and performance were compared between each athlete and the 19 other athletes with the closest performance. To ensure that those suffering deleterious health effects from dysnatremia would not bias outcomes, performance variables were also analyzed with those with dysnatremia removed. In this parallel analysis with normonatremic athletes, the same analysis of primary outcomes were analyzed using ANOVA, chi-squared, and Pearson’s product-moment correlation to identify a potential relationship between sodium consumption and performance. As heat stress is known to increase thirst, sweating, and compensatory hydration requirements [30], ambient temperature could presumably impact sodium intake and subsequently effect performance. The average daytime temperature was 92.8 °F (33.8 °C) for the hot races and 57.7 °F (14.2 °C) for the cold races [7], so race temperature both in absolute (average daytime temperature) and categorical (hot or cold) were included as a control variable in our analysis. To determine a potential difference in average temperature for sodium consumption groups, a Welch two-sample *t* test was used. For categorical temperature (hot or cold), a chi-squared test was used. In either case, the tests were leveraged to assess the null hypothesis that sodium consumption would not differ significantly based on race temperature. Acceptable type 1 error was set to 5% (alpha = 0.05) for interpretation of statistical tests, with 95% confidence intervals. Univariate correlations and relationships were analyzed with Pearson’s correlation coefficient and linear least squares regression. All analyses were performed with the statistical software R, version 4.0.

## Results

**Fig. 1:**
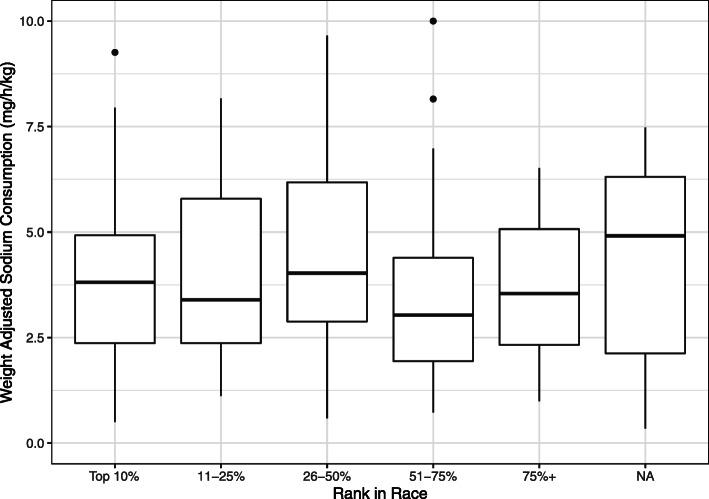
Weight-adjusted sodium intake versus performance from percentile based groupings. Sodium intake does not appear significantly correlated with rank-based performance. Extreme outliers in sodium consumption (four values > 10) were excluded to improve plot readability

There were 266 participants enrolled, 217 (82%) with complete sodium supplement rate data, 174 (80%) with post-race sodium measurements, and 161 (74%) with both pre-race weights and sodium ingestion data. There were no significant differences in demographic variables between the sodium ingestion groups other than the low sodium category, which had a larger body mass index and run fewer ultramarathons compared to the two other sodium groups (Table 1). Sodium supplementation per weight-based intake rate (mg/h/kg) did not have any statistically significant associations with hydration, dysnatremia, or race performance, among other variables (Table 2) This did not change with a non-weight-based analysis. [(mg/h), Supplementary Tables 1 and 2] When sodium intake rate was examined as a continuous variable, no significant associations were found with percentile rankings (Fig. 1).

**Table 1:** Demographics by weight-based sodium intake

Variable	Low sodium intake Mean (SD); Median (min–max)	Medium sodium intake Mean (SD); Median (min–max)	High sodium intake Mean (SD); Median (min–max)	*P* value
Runner characteristics, *n* (%)	53 (32.9)	53 (32.9)	55 (34.2)	–
Age, years	45 (8.4); 45 (24–63)	42 (8.3); 42 (23–56)	41 (9.2); 41 (23–65)	0.06
Sex				0.66
Female, *n* (%)	15 (28.3)	19 (35.9)	19 (35)	
Male, *n* (%)	38 (71.7)	34 (64.2)	35 (64.8)	
Height, cm	174 (8.8); 175 (157–188)	177 (8.1); 177 (158–193)	175 (8.2); 177 (160–195)	0.3
Weight (starting), kg	76 (13.4); 74 (52–106)	73 (10.9); 74 (58–101)	72 (10.5); 73 (52–94)	0.26
BMI, kg/m^2^	25 (3.3); 24 (18–34)	23 (2.4); 23 (19–31)	23 (2.7); 23 (18–33)	0.01
Pack weight (starting), kg	10 (2); 10 (7–17)	10 (1.7); 10 (8–15)	10 (1.8); 10 (6–15)	0.37
# prior marathons	13 (15.2); 7 (0–50)	14 (28.8); 3 (0–130)	7 (9.6); 3 (0–40)	0.15
# prior ultramarathons	6 (6.7); 5 (0–35)	11 (14.3); 5 (0–70)	6 (7.3); 4 (0–40)	0.02
Running distance/week, km	72 (35.4); 70 (20–175)	62 (31); 60 (16–160)	81 (93.6); 60 (25–700)	0.28
Greatest running distance/week, km	131 (75.8); 105 (18–300)	132 (79); 105 (20–300)	144 (133.1); 100 (24–700)	0.76
Longest single run	121 (109); 88 (14–700)	118 (83.3); 100 (12–400)	117 (72); 90 (30–330)	0.97

Percentage of missing values from variables of sex, height, pack weight, prior marathons, prior ultramarathons, and training data = < 5% of data

**Table 2:** Post-race analysis by weight-based sodium intake

Variable	Low sodium intake Mean (SD); Median (min–max)	Medium sodium intake Mean (SD); Median (min–max)	High sodium intake Mean (SD); Median (min–max)	*P* value
Runner characteristics, *n* (%)	53 (32.9)	53 (32.9)	55 (34.2)	–
Body weight change, kg	− 4 (7.9); − 2 (− 50–2)	− 2 (4.4); − 2 (− 14–3)	− 4 (5.5); − 2 (− 17–2)	0.24
Hydration				0.31
Dehydration, *n* (%)	15 (31.9)	10 (20)	17 (35.4)	
Euhydration, *n* (%)	18 (38.3)	23 (46)	22 (45.8)	
Overhydration, *n* (%)	14 (29.8)	17 (34)	9 (18.8)	
Serum sodium, mEq	141 (5.8); 140 (126–158)	140 (6.3); 140 (126–158)	142 (5.8); 140 (133–160)	0.38
Creatinine, mg/dL	1 (0.3); 1 (0.5–1.8)	1 (0.5); 1 (0.5–3.7)	1 (0.3); 1 (0.1–1.8)	0.62
Sodium intake rate, mg/h/kg	132 (55.5); 122 (25–235)	269 (66); 250 (175–450)	533 (260.7); 450 (267–1500)	< 0.01
Total sodium ingested, g	2 (1.2); 2 (0–6)	4 (1.6); 4 (1–9)	7 (3.6); 7 (3–18)	< 0.01
Sodium diagnoses				0.64
Hyponatremia, *n* (%)	3 (7.1)	4 (9.8)	1 (2.2)	
Hypernatremia, *n* (%)	10 (23.8)	8 (19.5)	9 (20)	
Normonatremia, *n* (%)	29 (69.1)	29 (70.7)	35 (77.8)	
Temperature of races				0.81
Hot races, *n* (%)	33 (62.3)	35 (66)	33 (60)	
Cold races, *n* (%)	20 (37.7)	18 (34)	22 (40)	
Pace, min/km	11 (3); 11 (6–18)	11 (3.2); 10 (6–20)	10 (3); 9 (7–18)	0.31
Total race time, h	15 (3.9); 15 (8–23)	14 (4.2); 14 (8–26)	13 (3.8); 12 (9–23)	0.28
Finishing rank, *n* (%)				0.35
Top 10%	7 (14.3)	8 (15.4)	7 (14.3)	
11–25%	8 (16.4)	8 (15.4)	12 (24.5)	
26–50%	9 (18.4)	13 (25)	17 (34.7)	
51–75%	16 (32.7)	15 (28.9)	6 (12.2)	
> 75%	9 (18.4)	8 (15.4)	7 (14.3)	

Percentage of missing values from pre-race weight = 26%, hydration = 33%, sodium and creatinine = 32%, and rank = 11% (89% finished the races)

**Fig. 2:**
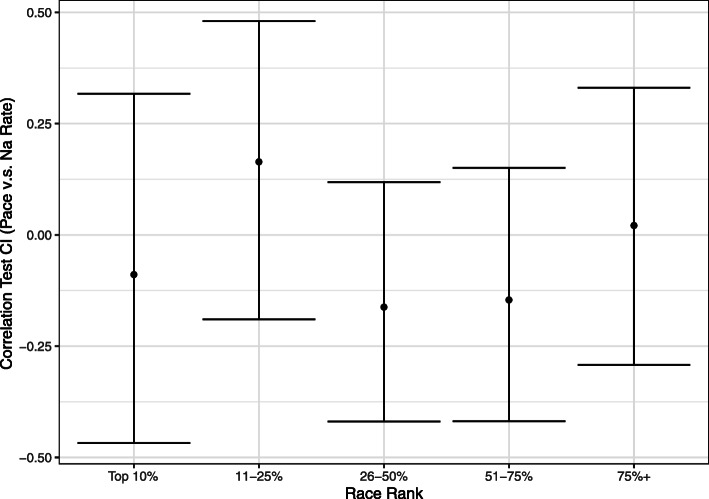
Pearson’s product moment-correlation coefficient and 95% confidence interval for pace and sodium intake rate controlled for similar paced runners using percentile based grouping.

Sodium intake in similarly paced runners was not significantly correlated with finishing quintile (Fig. 2). Controlling for dysnatremic runners, sub-group analysis of the 109 (68%) normonatremic participants found no significant differences in sodium intake and performance outcomes (Table 3).

**Table 3:** Post-race analysis by sodium intake in normonatremic participants

Variable	Low sodium intake Mean (SD); Median (min–max)	Medium sodium intake Mean (SD) Median (min–max)	High sodium intake Mean (SD); Median (min–max)	*P* value
Runner characteristics, *n* (%)	34 (31.2)	40 (36.7)	35 (32.1)	–
Body weight change, kg	− 3 (4.2); − 2 (− 14–2)	− 3 (5.1); − 2 (− 14–2)	− 5 (5.8); − 2 (− 16–2)	0.44
Hydration				
Dehydration, *n* (%)	10 (35.7)	11 (32.4)	11 (36.7)	0.82
Euhydration, *n* (%)	11 (39.3)	13 (38.2)	14 (46.7)	
Overhydration, *n* (%)	7 (25)	10 (29.4)	5 (16.7)	
Sodium intake rate, mg/h	135 (46.1); 129 (40–200)	266 (51.7); 250 (210–360)	577 (264.8); 495 (370–1500)	< 0.01
Total sodium ingested, g	2 (1); 2 (1–4)	4 (1.1) ; 4 (2–7)	8 (3.2); 7 (3–18)	< 0.01
Pace, min/km	11 (3.3); 11 (6–18)	11 (3); 9 (6–18)	11 (3.3); 10 (7–18)	0.85
Total race time, h	15 (4.3); 14 (8–23)	14 (3.8); 12 (8–23)	14 (4.2); 13 (9–23)	0.84
Finishing Rank, *n* (%)				
Top 10%	4 (12.1)	5 (12.8)	3 (8.8)	0.98
11–25 %	4 (12.1)	7 (18)	7 (20.6)	
26–50 %	9 (27.3)	11 (28.2)	10 (29.4)	
51–75%	9 (27.3)	8 (20.5)	6 (11.7)	
> 75%	7 (21.2)	8 (20.5)	8 (23.5)	

Percentage of missing values: hydration = 16%, and rank = 3%

**Fig. 3:**
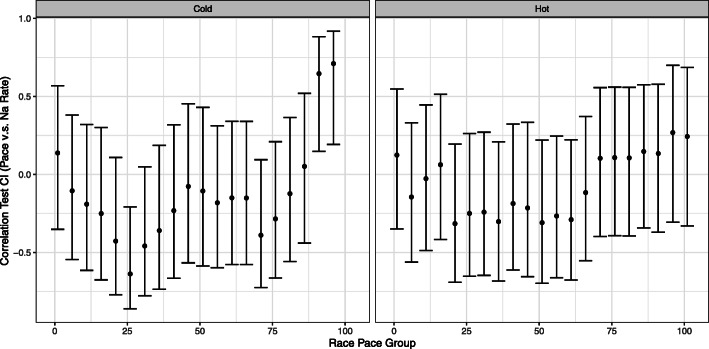
Similar paced runners correlated with pace versus sodium intake in hot versus cold climates. This plot demonstrates a lack of overwhelming evidence for correlation between sodium intake and performance at any performance level. Each race pace group corresponds to a sliding window of the 20 runners with the most similar overall race performance according to average pace. Pearson’s product moment correlation is used to assess linear relationship between pace and sodium intake. Each bar gives the correlation value and 95% confidence interval

There were 101 (56%) runners enrolled from hot races and 60 (44%) from cold races. Similarly paced participants were analyzed by sodium intake rate and separated by race temperatures, with no performance difference in hot versus cold environments (Fig. 3). When controlled for weight-adjusted sodium intake, performance was poorly correlated with hydration (*r* = − 0.152, 95% CI − 0.348–0.057). Dehydrated runners had improved performance compared to those overhydrated, with 11% of top 25th percentile finishers dehydrated (versus 2.8% overhydrated), with 3.6 min/km faster pace and 4.6 h faster race time (Table 4).

**Table 4:** Hydration by sodium intake

Variable	Dehydration Mean (SD); Median (min/max)	Euhydration Mean (SD); Median (min/max)	Overhydration Mean (SD); Median (min–max)	*P* value
Runner characteristics, *n* (%)	42 (29.1)	63 (43.8)	39 (27.1)	
Age, years	45 (7.4); 45 (28–63)	42 (8.8); 43 (23–65)	42 (9.5); 43 (26–57)	0.26
Sex				
Female, *n* (%)	16 (38)	16 (25)	12 (31)	0.38
Male, *n* (%)	26 (62)	47 (75)	27 (69)	
Height, cm	176 (7.7); 173 (164–193)	177 (8.4); 178 (158–189)	175 (9.2); 177 (157–195)	0.53
Weight (starting), kg	73 (13.2); 72 (52–103)	75 (10.9); 74 (55–105)	75 (11.3); 75 (57–107)	0.59
BMI, kg/m^2^	24 (2.9); 23 (18–31)	24 (2.7); 24 (20–33)	25 (3.3); 24 (19–34)	0.55
Pack weight (starting), kg	9 (2.1); 9 (6–15)	11 (2.8); 10 (7–17)	13 (3.4); 11 (8–16)	< 0.01
Body weight change, kg	− 10 (7.6); − 10 (− 50–3)	− 2 (0.8); − 2 (− 3–0)	1 (0.7); 1 (0–2)	< 0.01
Serum sodium, mEq	141 (4.3); 140 (133-153)	142 (6.8); 140 (124–160)	139 (5.4); 139 (126–156)	0.05
Sodium intake rate, mg/h	323 (198.6); 280 (74–860)	329 (264.3); 250 (40–1500)	279 (156.4); 240 (83–768)	0.52
Total sodium ingested, g	4 (2.4); 4 (1–9)	4 (3.5); 4 (1–18)	5 (3.1); 4 (1–14)	0.53
Sodium diagnoses				< 0.01
Hyponatremia, *n* (%)	1 (2.4)	1 (1.9)	6 (18.8)	
Hypernatremia, *n* (%)	8 (19.5)	14 (26.4)	4 (12.5)	
Normonatremia, *n* (%)	32 (78.1)	38 (71.7)	22 (68.8)	
Temperature of races				0.31
Hot races, *n* (%)	19 (45.2)	49 (77.8)	24 (60)	
Cold races, *n* (%)	23 (54.8)	14 (22.2)	14 (40)	
Pace, min/km	9 (2.1); 9 (6–14)	11 (2.8); 10 (7–18)	13 (3.4); 13 (6–20)	< 0.01
Total race time, h	12.4 (2.9); 12 (8–20)	14 (3.7); 13 (9–23)	17 (4.3); 18 (8–26)	< 0.01
Finishing rank, *n* (%)				< 0.01
Top 10%	8 (20)	9 (14.5)	4 (10)	
11–25%	8 (20)	18 (29)	0	
26–50%	14 (35)	17 (27.4)	6 (15)	
51–75%	7 (17.5)	13 (21)	14 (35)	
> 75%	3 (7.5)	5 (8.1)	16 (40)	

Percentage of missing values from: pre-race weight = 26%, hydration = 33%, sodium = 32%, and rank = 11% (89% finished the races)

## Discussion

This study found that neither the weight-based rate of sodium ingestion nor amount of sodium consumed over the duration of an 80-km (50 mi) ultramarathon was associated with race performance as measured by average pace, race completion time, or finishing rank. These findings were consistent with prior endurance running research [5, 6, 25, 26]. The total amount of ingested supplemental sodium of 2–7.2 g was comparable to prior studies on this subject [5, 6, 25, 26]. Our study’s primary outcome that sodium intake was not associated with running performance did not change when analyzed by similarly paced runners, controlled for dysnatremia, and when the runners were separated by extremes of temperature. This study provides further evidence that sodium supplementation is not an important contributor to ultramarathon race performance.

The main advantage of sodium supplementation—before, during, or after exercise—is plasma volume expansion [13], as fluids with sodium maintain plasma volume better than water alone during exercise [31]. However, we did not observe an impact of sodium intake rates on hydration levels. Rather, a strong inverse relationship has been seen between greater weight loss and improved performance in marathons [32], triathlons [33], adventure racing [34], and ultramarathons [27, 35, 36]. This was consistent with our observation that dehydrated runners, controlled for weight-adjusted sodium intake, out-performed the other runners. Conversely, those overhydrated ran slower and took over 4 h longer to complete the ultramarathon. It is theorized that loss of 3% total body weight represents physiologic euhydration, as this is the weight of water released with the oxidization of stored glycogen. It is reasonable that the larger glycogen stores used by faster runners resulting in better finishing times represent a portion of the weight loss used to define the hydration cohort. Sodium intake would not have a large impact on this relationship.

A popular misconception held by endurance athletes is that sodium supplementation will prevent the development of exercise-associated hyponatremia during endurance races [3]. Field data suggests that sodium supplementation does not prevent the development of hyponatremia during triathlons when athletes drink ad libitum [5, 6]. Sodium supplementation does, however, attenuate the decline in serum sodium when athletes drink to fully replace body weight losses [31], but does not prevent hyponatremia when athletes overdrink during exercise [2, 6, 7, 25, 31, 37]. The observed rates of dysnatremia and normonatremia were very similar between sodium intake groups, despite disparate amounts of both weight-based and total ingested sodium. The pseudo-axiom that sodium supplements can “protect” against hyponatremia could be dangerous, as excess sodium intake can trigger osmoreceptor stimulation and thirst symptoms [26], resulting in increased fluid intake [38]. While there has been no reported exercise-induced hypernatremic fatalities from either profound water losses or exuberant sodium intake, the minimum lethal dose of salt (extrapolated from pediatrics) is 0.75–3 g/kg body weight [39]. Our findings build on past investigations to help dispel the myth that sodium supplementation prevents the development of dilutional and/or symptomatic exercise-associated hyponatremia, which has been linked to over a dozen fatalities [8].

A limitation of these data are that sodium intake was calculated solely on the supplements ingested by the study participants. While the brand and sodium concentration of the supplements were confirmed by the researchers, exact amount of sodium ingested, dietary intake, sodium sweat rate, and plasma volume levels were not determined; driven by logistical limitations that was consistent with prior ultramarathon research [2, 40]. Exact amounts of sodium ingestion may have been inexact because of recall bias, but this was consistent across the entire study population. While weight was unable to be gathered on 26% of the study participants, this unlikely altered our conclusions as there was no difference in performance outcomes when sodium was analyzed as a non-weight-based predictor. Also, we did not gather information on injuries that could have impacted performance, and an injury log could provide further insight on this. To truly personalize sodium intake, sodium output could be quantified by sweat analysis and “matched” by intake, which warrants further investigation to determine if matched sodium intake could impact endurance running performance, particularly in events held in relatively hot conditions.

## Conclusions

This study suggests that ad libitum sodium supplements in ultramarathon runners do not have any significant association with ultramarathon performance. Lower sodium intake rate and amount was not associated with exercise-associated hyponatremia, and higher sodium was not associated with exercise-induced hypernatremia. Dehydrated runners were found to have the best performance, with significantly faster pace and finishing times. This reinforces the message to avoid overhydration and not to agressively pursue electrolyte supplements to impact running performance.   

## Supplementary Information

**Additional file 1:.** Data Collection Questionnaire

**Additional file 2: Supplementary Table 1**. Demographics by sodium intake rate

**Additional file 3: Supplement Table 2**. Post-race analysis by sodium intake rate

## Data Availability

The datasets used and analyzed during this current study are available from the corresponding author on reasonable request.

## Abbreviations

Km: Kilometers
Mi: Miles
Kcal: Kilocalories
Mg: Milligram
Kg: Kilogram
H: Hour
Mmol: Milimoles
L: Liter
Cm: Centimeters
M: Meters
mEq: Miliequivalents
dL: Decaliters
g: Grams
min: Minute
CI: Confidence intervals
Mmol: Milimoles
SD: Standard deviation
Min: Minimum
Max: Maximum
