# Supplementary material for: Prospective Observational Study of Weight-based Assessment of Sodium Supplements on Ultramarathon Performance (WASSUP)
Source: Sports Med Open. 2021 Feb 17;7:13. doi: 10.1186/s40798-021-00302-0 (PMC7886928; doi:10.1186/s40798-021-00302-0)
Supplement: Supplementary file 1 — Additional file 1:. Data Collection Questionnaire [file 40798_2021_302_MOESM1_ESM.doc]

**Prospective Observational Study of Weight-based Assessment of Sodium Supplements on Ultramarathon Performance (WASSUP)**

***Sports Medicine Open***

Grant S Lipman^1^, Tamara Hew-Butler^2^, Caleb Phillips^3^, Brian Krabak^4^, Patrick Burns^1^

^1^ Department of Emergency Medicine, Stanford University School of Medicine, Palo Alto, CA, USA; ^2^ Exercise and Sport Science, College of Education, Wayne State University, Detroit, MI, USA, ^3^ Computational Science, University of Colorado, Boulder, CO. USA; ^4^ Department of Orthopedics and Sports Medicine, University of Washington, Seattle, WA, USA

**Corresponding author:** Grant S. Lipman, MD. Department of Emergency Medicine, Stanford University School of Medicine. 900 Welch Rd, Suite #350, Palo Alto, CA. 94304, USA. [grantlip@hotmail.com](mailto:grantlip@hotmail.com) (415) 290-9286.

Data Collection Questionnaire

| **Pre-Race** | |
| --- | --- |
| Age: | Sex: M F |
| Height (cm): | Pack Weight (kg): |
| # Prior Marathons: | # Prior Ultras: |
| Average running distance / week: | Highest running distance in 1 week: |
| Longest single run: |
| During the long stage of the race, what is your hydration plan? (circle the 1 most appropriate)  1. Drink when thirsty  2. Drink the maximum you can (maximum tolerated)  3. Drink to a pre-determined schedule  4. Drink before you are thirsty?  5. Drink to urine color / amount of urine?  6. Other _____________________ | |
| During the long stage of the race, what type of electrolyte supplementation will you consume? (circle the 1 most appropriate)   1. Salt tabs 2. Mostly water with some electrolyte mix 3. Equal parts water and electrolyte mix 4. Mostly electrolyte mix 5. Salt tabs and electrolyte mix 6. Only water 7. Other _________________ | |
| What is the concentration (mg of sodium per dose) in your electrolyte supplement? | |
| What is your planned rate of consumption of electrolytes during the long stage?  (mg of sodium / hour) | |

| **Morning of Stage 5** |
| --- |
| Pack Weight (kg): |
| Body Weight (kg): |

| **After Stage 5** | | | |
| --- | --- | --- | --- |
| Did you have any nausea and / or vomiting during the long stage? Yes / No  If Yes, circle the most severe:   1. Almost non-existent 2. Mild 3. Moderate 4. Severe 5. Debilitating   Were you compliant with electrolyte supplementation plan?  Yes / No / Other_______  If No, did you take more or less (i.e. # of pills) then intended and by how much?  _________  Were you compliant with your hydration plan? Yes / No / Other________  What was the average color of your urine?   1. No urine 2. Brown 3. Dark Yellow 4. Golden Yellow 5. Straw 6. Very light | | | |
| Body Weight (kg): | | Take NSAID during Stage 5? Yes / No  If Yes, mg ingested (approximately) | |
| Na: | BUN: | | Cr: |
| Time (per checkpoint): | | | |
