# Supplementary material for: Prospective Observational Study of Weight-based Assessment of Sodium Supplements on Ultramarathon Performance (WASSUP)
Source: Sports Med Open. 2021 Feb 17;7:13. doi: 10.1186/s40798-021-00302-0 (PMC7886928; doi:10.1186/s40798-021-00302-0)
Supplement: Supplementary file 2 — Additional file 2: Supplementary Table 1. Demographics by sodium intake rate [file 40798_2021_302_MOESM2_ESM.docx]

**Prospective Observational Study of Weight-based Assessment of Sodium Supplements on Ultramarathon Performance (WASSUP)**

***Sports Medicine Open***

Grant S Lipman^1^, Tamara Hew-Butler^2^, Caleb Phillips^3^, Brian Krabak^4^, Patrick Burns^1^

^1^ Department of Emergency Medicine, Stanford University School of Medicine, Palo Alto, CA, USA; ^2^ Exercise and Sport Science, College of Education, Wayne State University, Detroit, MI, USA, ^3^ Computational Science, University of Colorado, Boulder, CO. USA; ^4^ Department of Orthopedics and Sports Medicine, University of Washington, Seattle, WA, USA

**Corresponding author:** Grant S. Lipman, MD. Department of Emergency Medicine, Stanford University School of Medicine. 900 Welch Rd, Suite #350, Palo Alto, CA. 94304, USA. [grantlip@hotmail.com](mailto:grantlip@hotmail.com) (415) 290-9286.

Supplementary Table 1. Demographics by sodium intake rate

.

| Variable | Low Sodium Intake  Mean (SD) | Medium Sodium Intake  Mean (SD) | High Sodium Intake  Mean (SD) | P value |
| --- | --- | --- | --- | --- |
| Runner characteristics, n (%) | 75 (34.6) | 84 (38.7) | 58 (26.7) |  |
| Age, years | 41 (9.3) | 42 (8.2) | 41 (9.3) | 0.72 |
| Sex  Female, n (%)  Male, n (%) | 30 (40)  45 (60) | 34 (40)  50 (60) | 18 (31)  40 (69) | 0.46 |
| Height, cm | 173 (8.2) | 175 (8.7) | 177 (8.7) | 0.02 |
| Weight (starting), kg | 72 (11.9) | 73 (11.7) | 76 (11.1) | 0.12 |
| BMI, kg/m^2^ | 24 (3.2) | 24 (2.8) | 24 (2.6) | 0.53 |
| Pack weight (starting), kg | 10 (2) | 10 (1.7) | 10 (2.5) | 0.37 |
| # prior marathons | 15 (20.7) | 11 (21.5) | 6 (7.9) | 0.04 |
| # prior ultramarathons | 7 (11.1) | 9 (10.6) | 6 (6.8) | 0.11 |
| Running distance / week, km | 70 (34.2) | 58 (27.7) | 76 (91) | 0.16 |
| Greatest running distance / week, km | 147 (82.9) | 143 (96.7) | 149 (125.9) | 0.94 |
| Longest single run, km | 116 (101.5) | 113 (86.3) | 118 (69.5) | 0.95 |

Percentage of missing values from variables of sex, height, pack weight, prior marathons, prior ultramarathons, and training data = < 5% of data.
