# Supplementary material for: Prospective Observational Study of Weight-based Assessment of Sodium Supplements on Ultramarathon Performance (WASSUP)
Source: Sports Med Open. 2021 Feb 17;7:13. doi: 10.1186/s40798-021-00302-0 (PMC7886928; doi:10.1186/s40798-021-00302-0)
Supplement: Supplementary file 3 — Additional file 3: Supplement Table 2. Post-race analysis by sodium intake rate [file 40798_2021_302_MOESM3_ESM.docx]

**Prospective Observational Study of Weight-based Assessment of Sodium Supplements on Ultramarathon Performance (WASSUP)**

***Sports Medicine Open***

Grant S Lipman^1^, Tamara Hew-Butler^2^, Caleb Phillips^3^, Brian Krabak^4^, Patrick Burns^1^

^1^ Department of Emergency Medicine, Stanford University School of Medicine, Palo Alto, CA, USA; ^2^ Exercise and Sport Science, College of Education, Wayne State University, Detroit, MI, USA, ^3^ Computational Science, University of Colorado, Boulder, CO. USA; ^4^ Department of Orthopedics and Sports Medicine, University of Washington, Seattle, WA, USA

**Corresponding author:** Grant S. Lipman, MD. Department of Emergency Medicine, Stanford University School of Medicine. 900 Welch Rd, Suite #350, Palo Alto, CA. 94304, USA. [grantlip@hotmail.com](mailto:grantlip@hotmail.com) (415) 290-9286.

Supplement Table 2. Post-race analysis by sodium intake rate

| Variable | Low Sodium Intake  Mean (SD) | Medium Sodium Intake  Mean (SD) | High Sodium Intake  Mean (SD) | P value |
| --- | --- | --- | --- | --- |
| Runner characteristics, n (%) | 75 (34.6) | 84 (38.7) | 58 (26.7) | - |
| Body weight change, kg | -4 (8) | -3 (4.3) | -4 (5.5) | 0.31 |
| Hydration  Dehydration, n (%)  Euhydration, n (%)  Overhydration, n (%) | 15 (31.3)  29 (31.3)  13 (27.1) | 13 (24.1)  23 (42.6)  18 (33.3) | 14 (32.6)  20 (46.5)  9 (20.9) | 0.7 |
| Serum sodium, mEq | 141.5 (6.3) | 140.4 (5.8) | 140.6 (5.6) | 0.62 |
| Creatinine, mg/dL | 1 (0.3) | 1 (0.5) | 1 (0.3) | 0.7 |
| Sodium intake rate (mg/hr) | 131 (51.4) | 277 (55.8) | 571 (248.5) | <0.01 |
| Total sodium ingested (g) | 2 (0.9) | 4 (1.2) | 8 (3.2) | < 0.01 |
| Sodium diagnoses  Hyponatremia, n (%)  Hypernatremia, n (%)  Normonatremia, n (%) | 4 (8)  12 (24)  34 (68) | 3 (5.6)  11 (20.4)  40 (74.1) | 3 (6.8)  6 (13.6)  35 (79.6) | 0.74 |
| Temperature of races  Hot races, n (%)  Cold races, n (%) | 45 (60)  30 (40) | 44 (52)  40 (47.6) | 34 (57.6)  25 (42.4) | 0.61 |
| Pace, min/km | 11 (3.1) | 11 (3.1) | 11 (3.2) | 0.99 |
| Total race time, hr | 14 (3.9) | 14 (4) | 14 (4) | 1 |
| Finishing Rank, n (%)  Top 10%  11 – 25 %  26 – 50 %   1. – 75%   >75% | 9 (13.6)  15 (22.7)  12 (18.2)  18 (27.3)  12 (18.2) | 9 (11.8)  8 (10.5)  25 (32.9)  19 (25)  15 (19.7) | 7 (13.2)  10 (18.9)  14 (26.4)  9 (17)  13 (24.5) | 0.4 |

Percentage of missing values from: pre-race weight = 26%, hydration = 33%, sodium and creatinine = 32%, and rank = 11% (89% finished the races).
